# Supplementary material for: A pathway towards high-quality development of the manufacturing industry: Does scientific and technological talent matter?
Source: PLoS One. 2024 Mar 18;19(3):e0294873. doi: 10.1371/journal.pone.0294873 (PMC10947903; doi:10.1371/journal.pone.0294873)
Supplement: Attachment — Submitted filename: Review Comments.pdf [file pone.0294873.s002.pdf]

The manuscript entitled “Study on the impact of scientific and technological talents on high-quality development of manufacturing industry: An empirical study based on provincial panel data” provides an empirical analysis on studying the relationship between scientific and technological talents and high-quality development of manufacturing industry, using the two-way fixed effects model and the spatial econometric model as the identification strategies. Also, the authors further explore two underlying mechanisms through which scientific and technological talents can affect high-quality development of manufacturing industry, i.e., consumption structure upgrading and productive service industry aggregation. Overall, the manuscript is interesting. I have several minor comments and suggestions for the authors:

1. The title of the article is too lengthy, resulting in poor comprehensibility. Thus, I would recommend the authors to modify the title of the manuscript as “A pathway towards high-quality development of manufacturing industry: Does scientific and technological talent matter?”

2. The abstract should provide a highly concise introduction to the research background, methods, findings, and significance of the article. However, I cannot see these items clearly in the current version. Also, the research gap is not identified in a compelling way in the introduction section.

3. English language and grammar issues are too abundant throughout the manuscript, and need a professional proofreading. For example, see the following sentence:
“Based on human capital theory, endogenous growth theory, and industrial agglomeration theory to construct a theoretical analysis framework, panel data from 30 provinces in China from 2012 to 2021 were used as research samples to measure the level of scientific and technological talents and the level of high-quality development of the manufacturing industry by the entropy weight method, and a two-way fixed effect model, a mediated effect model, and a spatial Durbin model were used to empirically investigate the influence of scientific and technical talents on the high-quality

development of the manufacturing industry”

This is only one example, and similar issue are abundant.

4. Comparisons with other studies have to be provided in the DISCUSSION section. Please interpret and describe the significance of your findings in light of what was already known about the research problem being investigated and explain any new understanding or fresh insights about the problem after you've taken the findings into consideration. Please provide a comparison with other studies.

5. Its topic appears to be within the scope of carbon finance or sustainable finance, the authors should cite research from *PLOS ONE* journal to connect to this ongoing discourse.

6. The last section needs to be re-organized, with more insightful implications of your findings for managers and researchers (ideally in two separate sections, i.e., theoretical and practical implications). Also, please elaborate on more limitations and future research directions.

Good luck!
