# Supplementary material for: A pathway towards high-quality development of the manufacturing industry: Does scientific and technological talent matter?
Source: PLoS One. 2024 Mar 18;19(3):e0294873. doi: 10.1371/journal.pone.0294873 (PMC10947903; doi:10.1371/journal.pone.0294873)
Supplement: Attachment — Submitted filename: Response to Reviewers.docx [file pone.0294873.s003.docx]

**Response to Reviewers’**

We would like to thank the reviewers for their comments, which we have reflected on repeatedly and revised the article accordingly. The following is the response to the reviewers' and journals' comments.

**Response to journal comments：**

1. Please ensure that your manuscript meets PLOS ONE's style requirements, including those for file naming.

**Response:** Based on the journal's template, We have made the first changes to the manuscript's typography, fonts, and file name.

2. Please remove any funding-related text from the manuscript and let us know how you would like to update your Funding Statement.

**Response:** Changes have been made to the Acknowledgments section of the manuscript by removing the reference to funding information. In addition, no changes are required to the Funding Statement of this paper, which we have explained in the cover letter.

3. Please state what role the funders took in the study. If the funders had no role, please state: "The funders had no role in study design, data collection and analysis, decision to publish, or preparation of the manuscript." If this statement is not correct you must amend it as needed. Please include this amended Role of Funder statement in your cover letter; we will change the online submission form on your behalf.

**Response:** Concerning the role of funders in the financial disclosure section, funders have a role in the study, and we have described the role of funders in the cover letter.

4. We note that you have stated that you will provide repository information for your data at acceptance. Should your manuscript be accepted for publication, we will hold it until you provide the relevant accession numbers or DOIs necessary to access your data. If you wish to make changes to your Data Availability statement, please describe these changes in your cover letter and we will update your Data Availability statement to reflect the information you provide.

**Response:** We do not need to change the data availability statement.

5. PLOS requires an ORCID iD for the corresponding author in Editorial Manager on papers submitted after December 6th, 2016. Please ensure that you have an ORCID iD and that it is validated in Editorial Manager. To do this, go to ‘Update my Information’ (in the upper left-hand corner of the main menu), and click on the Fetch/Validate link next to the ORCID field. This will take you to the ORCID site and allow you to create a new iD or authenticate a pre-existing iD in Editorial Manager.

**Response:** We have made corrections to the personal information as prompted by you by linking ORCID iD to my Editorial Manager account.

6. Please amend the manuscript submission data (via Edit Submission) to include author Dan LI.

**Response:** We have corrected the submission information as you requested by adding the author Li Dan.

7. Please remove your figures from within your manuscript file, leaving only the individual TIFF/EPS image files, uploaded separately.

**Response:** We removed the icons from the manuscript and uploaded the icons in TIF format.

8. We note you have included a table to which you do not refer in the text of your manuscript. Please ensure that you refer to Table 2, 4, 14 and 15 in your text; if accepted, production will need this reference to link the reader to the Table.

**Response:** The tables in the manuscript have all been mentioned in the text, and the revised parts have been marked in red in the manuscript.

9. Please include captions for your Supporting Information files at the end of your manuscript, and update any in-text citations to match accordingly.

**Response:** We have added the supporting information section at the end of the article and uploaded the relevant documents.

**Responses to reviewers' comments：**

1. The title of the article is too lengthy, resulting in poor comprehensibility. Thus, I would recommend the authors to modify the title of the manuscript as “A pathway towards high-quality development of manufacturing industry: Does scientific and technological talent matter?”

**Response:** The title of the article has been revised in accordance with the recommendations of the reviewers.

2. The abstract should provide a highly concise introduction to the research background, methods, findings, and significance of the article. However, I cannot see these items clearly in the current version. Also, the research gap is not identified in a compelling way in the introduction section.

**Response:** In the abstract section, the content was revised to present the background, methodology, results, and significance of the article. In the introduction section, the limitations of the existing research and the contributions of the article were presented in the last two paragraphs of the introduction, respectively, highlighting the gaps and innovations between the article and the existing research results.

3. English language and grammar issues are too abundant throughout the manuscript, and need a professional proofreading.

**Response:** We have professionally proofread the entire article to correct any grammatical problems.

4. Comparisons with other studies have to be provided in the DISCUSSION section. Please interpret and describe the significance of your findings in light of what was already known about the research problem being investigated and explain any new understanding or fresh insights about the problem after you've taken the findings into consideration. Please provide a comparison with other studies.

**Response:** In the discussion section, a comparison of the results of this study with other studies is included, and the significance of the findings and the understanding of the research questions are elaborated, as described in the concluding sections of articles 4.1, 5.1, 5.2 and 5.3.

5. Its topic appears to be within the scope of carbon finance or sustainable finance, the authors should cite research from PLOS ONE journal to connect to this ongoing discourse.

**Response:** We have included in the references articles from PLOS ONE journals in the relevant categories.

6. The last section needs to be re-organized, with more insightful implications of your findings for managers and researchers (ideally in two separate sections, i.e., theoretical and practical implications). Also, please elaborate on more limitations and future research directions.

**Response:** We have reorganized the last part of the article to include the significance of the findings and the limitations and future research directions of the article. See articles 6.2 and 6.4 for details.

The modifications have been highlighted in red in document “Revised Manuscript with Track Changes”.
